# Supplementary material for: Patterns of Intron Gain and Loss in Fungi
Source: PLoS Biol. 2004 Nov 30;2(12):e422. doi: 10.1371/journal.pbio.0020422 (PMC532390; doi:10.1371/journal.pbio.0020422)
Supplement: Table S1 — Also available at http://genes.mit.edu/NielsenEtAl/. (4.3 MB ZIP). [file pbio.0020422.st001.zip › NielsenEtAl/html/613.html]

AN4475.1.NCU02509.1.MG07753.1.FG01081.1


```
 CLUSTAL W (1.82) Multiple Sequence Alignments - Introns Inserted


Sequence 1: AN4475.1	175 aa
Sequence 2: FG01081.1	173 aa
Sequence 3: NCU02509.1	170 aa
Sequence 4: MG07753.1	139 aa
Alignment Length: 176 aa
Number Identitical Residues: 106 aa
Alignment Score (without introns) 4162


MG07753.1 	-~-M0SSE~KTQNPMRELKIQKLVLNISVGESGDRLTRAAKVLEQLSGQTPVYS1KARYT
NCU02509.1	-~--~--M0KAQNPMRELRIQKLVLNICVGESGDRLTRAAKVLEQLSGQTPVYS1KARYT
FG01081.1 	-~-M~ASE~KANNPMRELKIQKLVLNISVGESGDRLTRAAKVLEQLSGQTPVYS1KARYT
AN4475.1  	M0SE~AKD~KSANPMRELRVQKLVLNISVGESGDRLTRAAKVLEQLSGQTPVYS1KARYT
          	  :  :.  *: ******::*******.************************** *****

MG07753.1 	VRTFGIRRNEKIAVHVTVRGPK~AEEILERGLKVKEYELRKRNFSETGNFGFGISEHIDL
NCU02509.1	VRTFGIRRNEKISVHVTVRGPK~AEEILERGLKVKEYELRKRNFSETGNFGFGISEHIDL
FG01081.1 	VRTFGIRRNEKIAVHVTVRGPK0AEEILERGLKVKEYELRKRNFSETGNFGFGISEHIDL
AN4475.1  	VRTFGIRRNEKIAVHVTVRGPK~AEEILERGLKVKEYELRKRNFSETGNFGFGISEHIDL
          	************:********* *************************************

MG07753.1 	GIN2VSPAAAAARAESVPAT~RSTASRP--------------------------------
NCU02509.1	GIN2VSPAAAAPRAESVPPT~ASPATTPSSGSSLAS-MPSSAKLPHYFLKRIGCYSLATW
FG01081.1 	GIK~YDPSIGIYGMDFYCCM~TRPGERVTRRRRTKSKIGASHRIKREETVKWFKSRFDGI
AN4475.1  	GIK~YDPNIGIYGMDFYCCM2TRPGERVAKRRRCKSTIGASHRINQTETMRWFKQRFDGI
          	**:  .*  .    :        ..   :      :.  ::                   

MG07753.1 	---
NCU02509.1	WRI
FG01081.1 	VR-
AN4475.1  	IR-
          	
```
